# Supplementary material for: Coordination Polymers Based on Rhenium Octahedral Chalcocyanide Cluster Anions and Ag+ Cations with Bipyridine Analogs
Source: Molecules. 2022 Nov 9;27(22):7684. doi: 10.3390/molecules27227684 (PMC9698110; doi:10.3390/molecules27227684)
Supplement: Supplementary file 1 [file molecules-27-07684-s001.zip › molecules-1999941-supplementary.pdf]

Electronic supplementary information

Coordination polymers based on rhenium octahedral chalcocyanide cluster and Ag^+ cations with bipyridine derivatives

Yulia M. Litvinova,[a] Yakov M. Gayfulin, [a,] Taisiya S. Sukhikh Konstantin A. Brylev[a] and Yuri V. Mironov[a]*

[a]Nikolaev Institute of Inorganic Chemistry SB RAS, 630090, 3, Acad. Lavrentiev ave., Novosibirsk, Russia.

Table of contents:

- **Table S1.** Crystal data and structure refinement for **1-6**: pp. 2S-2S;
- **Table S2.** Selected interatomic distances of compounds **1–6**: p. 4S;
- **Tables S3–S8.** Selected bond distances (Å) and angles (°) for **1-6**: pp. 5S–12S;

Table S1. Crystal data and structure refinement for 1-6.

Identification code	**1**	**2**	**3**	**4**	**5**	**6**
Empirical formula	$C_{54}H_{32}Ag_4N_{14}Re_6Se_8$	$C_{54}H_{32}Ag_4N_{14}Re_6S_8$	$C_{22}H_{12}Ag_4N_{14}Re_6Se_8$	$C_{28}H_{19}Ag_4N_{11}Re_6Se_8$	$C_{98}H_{74}Ag_4N_{16}O_2Re_6Se_8$	$C_{97.89}H_{73.96}Ag_4N_{15.94}O_{2.06}Re_6S_8$
Formula weight	3057.29	2682.09	2652.82	2689.90	3688.06	3311.60
Temperature/K	150(2)	150(2)	150(2)	150(2)	150(2)	150(2)
Crystal system	triclinic	triclinic	orthorhombic	monoclinic	monoclinic	monoclinic
Space group	P-1	P-1	Pbca	C2/m	$P2_1/c$	$P2_1/c$
a/Å	10.5725(7)	10.7136(3)	14.5198(3)	15.6758(3)	20.0889(5)	20.0626(7)
b/Å	11.2072(8)	11.0306(3)	15.5943(3)	11.5882(2)	12.4770(3)	12.4266(3)
c/Å	13.7913(9)	13.5130(4)	17.9669(3)	13.1195(3)	20.3997(5)	20.2079(6)
α/°	106.617(2)	105.7760(10)	90	90	90	90
β/°	95.128(2)	94.8770(10)	90	112.0250(10)	99.653(2)	100.7180(10)
γ/°	106.227(2)	106.6780(10)	90	90	90	90
Volume/Å^3	1477.81(17)	1448.92(7)	4068.18(13)	2209.29(8)	5040.8(2)	4950.1(3)
Z	1	1	4	2	2	2
ρ_{calc}g/cm^3	3.435	3.074	4.331	4.044	2.427	2.219
μ/mm^{-1}	18.503	14.135	26.848	24.720	10.874	8.301
F(000)	1364.0	1220.0	4608.0	2348.0	3392.0	3102.0
Crystal	0.12 × 0.09 ×	0.08 × 0.03 ×	0.08 × 0.04 ×	0.1 × 0.025 ×	0.23 × 0.2 ×	0.2 × 0.06 ×

size/mm^3	0.06	0.03	0.04	0.025	0.2	0.01
Radiation	MoKα (λ = 0.71073)	MoKα (λ = 0.71073)	MoKα (λ = 0.71073)	MoKα (λ = 0.71073)	MoKα (λ = 0.71073)	MoKα (λ = 0.71073)
2Θ range for data collection/°	4.206 to 63.07	4.032 to 61.096	4.454 to 63.012	4.496 to 61.016	4.05 to 57.178	3.874 to 61.016
Index ranges	-15 ≤ h ≤ 15, -16 ≤ k ≤ 16, -20 ≤ l ≤ 20	-15 ≤ h ≤ 15, -15 ≤ k ≤ 14, -19 ≤ l ≤ 19	-21 ≤ h ≤ 15, -22 ≤ k ≤ 22, -26 ≤ l ≤ 21	-22 ≤ h ≤ 21, -16 ≤ k ≤ 16, -18 ≤ l ≤ 18	-26 ≤ h ≤ 23, -16 ≤ k ≤ 15, -27 ≤ l ≤ 14	-28 ≤ h ≤ 28, 0 ≤ k ≤ 17, 0 ≤ l ≤ 28
Reflections collected	30938	27140	25418	13500	23623	15086
Independent reflections	9861 [R_{int} = 0.0498, R_{sigma} = 0.0503]	8886 [R_{int} = 0.0405, R_{sigma} = 0.0440]	6725 [R_{int} = 0.0430, R_{sigma} = 0.0400]	3524 [R_{int} = 0.0305, R_{sigma} = 0.0286]	11102 [R_{int} = 0.0241, R_{sigma} = 0.0345]	15086 [R_{int} = 0.0402, R_{sigma} = 0.0400]
Data/restraints /parameters	9861/0/388	8886/9/388	6725/6/254	3524/27/158	11102/0/599	15086/0/592
Goodness-of-fit on F^2	1.057	1.034	1.032	1.018	1.051	1.195
Final R indexes [I>=2σ (I)]	R_1 = 0.0279, wR_2 = 0.0646	R_1 = 0.0295, wR_2 = 0.0531	R_1 = 0.0289, wR_2 = 0.0514	R_1 = 0.0213, wR_2 = 0.0417	R_1 = 0.0283, wR_2 = 0.0674	R_1 = 0.0449, wR_2 = 0.1091
Final R indexes [all data]	R_1 = 0.0334, wR_2 = 0.0666	R_1 = 0.0374, wR_2 = 0.0564	R_1 = 0.0402, wR_2 = 0.0550	R_1 = 0.0283, wR_2 = 0.0437	R_1 = 0.0366, wR_2 = 0.0703	R_1 = 0.0540, wR_2 = 0.1121
Largest diff. peak/hole / e Å^{-3}	1.96/-2.44	1.58/-1.20	1.65/-1.57	1.25/-2.36	2.14/-1.66	3.08/-2.01

Table S2. Selected bond distances (Å)

	1	2	3	4	5	6
Re–Re	2.6315(2)–2.6405(2) / 2.635(3)	2.6014(3)–2.6047(3) / 2.603(1)	2.4783(3)–2.7365(3) / 2.6(1)	2.6176(3)–2.6426(3) / 2.62(8)	2.6223(3)–2.6476(3) / 2.629(6)	2.591(4)–2.6162(4) / 2.603(5)
Re–Se/Re–S	2.5055(4)–2.5294(4) / 2.521(7)	2.3922(13)–2.4121(13) / 2.406(5)	2.346(5)–2.6384(6) / 2.5(1)	2.511(4)–2.5783(6) / 2.52(2)	2.5154(5)–2.5328(5) / 2.521(5)	2.399(2)–2.420(2) / 2.408(6)
Re–C	2.097(4)–2.112(4) / 2.105(6)	2.107(6)–2.143(6) / 2.12(1)	2.041(5)–2.157(6) / 2.5(1)	2.094(4)–2.100(6) / 2.097(3)	2.088(5)–2.107(5) / 2.096(8)	2.099(8)–2.118(8) / 2.11(8)
Ag–N	2.139(3)–2.673(5) / 2.3(1)	2.133(5)–2.505(5) / 2.3(1)	1.994(5)–2.583(5) / 2.1(2)	2.140(3)–2.473(5) / 2.2(1)	2.176(4)–2.63(4) / 2.3(2)	2.164(7)–2.622(8) / 2.3(2)

Table S3. Selected bond distances (Å) and angles (°) for **1**

1	
Ag1–Ag1	3.2532(7)
Ag1–N1	2.258(4)
Ag1–N101	2.316(3)
Ag1–N102	2.444(4)
Ag2–N3	2.139(3)
Ag2–N201	2.319(4)
Ag2–N2	2.374(4)
Ag2–N202	2.394(4)
N1–Ag1–N101	169.17(13)
N101–Ag1–Ag1	136.17(9)
N102–Ag1–Ag1	152.53(9)
N101–Ag1–N102	70.35(12)
N1–Ag1–N102	98.84(12)
N1–Ag1–Ag1	54.46(9)
N2–Ag2–N202	103.16(12)
N201–Ag2–N2	94.88(13)
N3–Ag2–N2	107.04(14)
N201–Ag2–N202	71.42(12)
N3–Ag2–N201	149.61(13)
N3–Ag2–N202	121.51(14)
C1–N1–Ag1	135.9(3)
C101–N101–Ag1	124.8(3)
C112–N101–Ag1	117.1(3)
C110–N102–Ag1	126.7(3)
C111–N102–Ag1	112.8(3)
C2–N2–Ag2	162.2(3)
C201–N201–Ag2	124.6(3)
C212–N201–Ag2	116.0(3)
C210–N202–Ag2	126.5(3)

C211–N202–Ag2	114.4(3)
C3–N3–Ag2	156.1(3)

Table S4. Selected bond distances (Å) and angles (°) for **2**

2	
Ag1–Ag1	3.3081(8)
Ag1–N1	2.341(5)
Ag1–N101	2.400(5)
Ag1–N102	2.333(5)
Ag2–N3	2.407(5)
Ag2–N201	2.311(5)
Ag2–N2	2.133(5)
Ag2–N202	2.390(5)
N1–Ag1–N101	100.29(15)
N101–Ag1–Ag1	147.85(11)
N102–Ag1–Ag1	137.57(11)
N102–Ag1–N101	70.94(15)
N102–Ag1–N1	168.52(16)
N1–Ag1–Ag1	44.98(10)
N2–Ag2–N202	122.09(16)
N2–Ag2–N201	149.79(17)
N2–Ag2–N3	106.10(17)
N201–Ag2–N202	72.03(15)
N201–Ag2–N3	94.10(15)
N202–Ag2–N3	104.51(15)
C1–N1–Ag1	130.4(4)
C101–N101–Ag1	126.5(4)

C112–N101–Ag1	113.2(3)
C110–N102–Ag1	126.4(4)
C111–N102–Ag1	116.1(3)
C2–N2–Ag2	161.2(4)
C201–N201–Ag2	125.6(4)
C212–N201–Ag2	116.7(3)
C210–N202–Ag2	127.2(4)
C211–N202–Ag2	113.7(3)
C3–N3–Ag2	162.6(4)

Table S5. Selected bond distances (Å) and angles (°) for **3**

**3**	
Ag1–N1	2.330(5)
Ag1–N3	2.206(5)
Ag1–N11	2.462(5)
Ag1–N12	2.493(6)
Ag1–N3	2.206(5)
Ag2–N3	1.994(5)
Ag2–N1	2.426(6)
Ag2–N12	2.240(6)
Ag2–N3	1.994(5)
Ag3–C2	2.491(5)
Ag3–Se4	2.8665(7)
Ag3–Se3	3.0418(7)
Ag3–N1	2.309(5)
Ag3–N21	2.387(5)
Ag3–N22	2.583(5)
Ag3–Se3	3.0418(7)
Ag3–Se4	2.8665(7)
Ag1–N1	2.330(5)
Ag1–N3	2.206(5)

Ag1–N11	2.462(5)
Ag1–N12	2.493(6)
Ag1–N3	2.206(5)
Ag2–N3	1.994(5)
Ag2–N1	2.426(6)
Ag2–N12	2.240(6)
Ag2–N3	1.994(5)
Ag3–C2	2.491(5)
Ag3–Se4	2.8665(7)
Ag3–Se3	3.0418(7)
Ag3–N1	2.309(5)
Ag3–N21	2.387(5)
Ag3–N22	2.583(5)
Ag3–Se3	3.0418(7)
Ag3–Se4	2.8665(7)
Ag1–C3–N3	132.8(5)
Ag2–C3–N3	138.6(6)
Ag1–C3–N3	132.8(5)
Ag2–C3–N3	138.6(6)
Ag3–C13–N22	124.3(4)
Ag3–C13–N22	124.3(4)
Ag3–C12–N22	126.4(5)
Ag3–C12–N22	126.4(5)
Ag3–C15–N21	117.9(5)
Ag3–C15–N21	117.9(5)
Ag3–C14–N21	122.5(4)
Ag3–C14–N21	122.5(4)
Ag1–C13–N12	118.9(4)
Ag2–C13–N12	126.6(5)
Ag1–C13–N12	118.9(4)
Ag2–C13–N12	126.6(5)
Ag1–C10–N12	121.2(6)

Ag2–C10–N12	113.5(6)
Ag1–C10–N12	121.2(6)
Ag2–C10–N12	113.5(6)
Ag1–C17–N11	120.8(5)
Ag1–C17–N11	120.8(5)
Ag1–C14–N11	129.1(4)
Ag1–C14–N11	129.1(4)
Ag1–C1–N1	119.1(4)
Ag2–C1–N1	107.2(5)
Ag3–C1–N1	141.9(4)
Ag1–C1–N1	119.1(4)
Ag2–C1–N1	107.2(5)
Ag3–C1–N1	141.9(4)
Ag3–N2–C2	93.8(5)
Ag3–N2–C2	93.8(5)

Table S6. Selected bond distances (Å) and angles (°) for **4**

**4**	
Ag1–N11	2.233(3)
Ag1–N11	2.233(3)
Ag1–N1	2.473(5)
Ag2–N2	2.140(3)
Ag2–C1	2.503(5)
Ag2–Se4	2.7831(8)
Ag2–C1	2.503(5)
Ag2–N2	2.140(3)
Ag2–Se4	2.7831(8)
Ag1–N11	2.233(3)
Ag1–N11	2.233(3)
Ag1–N1	2.473(5)
Ag2–N2	2.140(3)

Ag2–C1	2.503(5)
Ag2–Se4	2.7831(8)
Ag2–C1	2.503(5)
Ag2–N2	2.140(3)
Ag2–Se4	2.7831(8)
N11–Ag1–N1	92.81(9)
N11–Ag1–N11	169.72(17)
N11–Ag1–N1	92.81(9)
C1–Ag2–Se4	85.64(13)
N2–Ag2–Se4	100.45(10)
N2–Ag2–N2	139.0(2)
N2–Ag2–C1	108.22(10)
N2–Ag2–Se4	100.45(10)
N2–Ag2–C1	108.22(10)
N1–C1–Re1	174.5(5)
N1–C1–Ag2	90.1(4)
C1–N1–Ag1	166.0(5)
C11–N11–Ag1	124.0(3)
C15–N11–Ag1	119.8(3)
C2–N2–Ag2	153.3(3)

Table S7. Selected bond distances (Å) and angles (°) for **5**

5	
Ag2–Ag2	3.3745(9)
Ag2–N2	2.630(4)
Ag2–N22	2.180(4)
Ag2–N21	2.176(4)
Ag1–N1	2.489(4)
Ag1–N11	2.207(4)

Ag1–Se1	3.0564(6)
Ag1–N12	2.214(4)
N2–Ag2–Ag2	126.58(10)
N22–Ag2–Ag2	74.78(12)
N22–Ag2–N2	90.73(14)
N21–Ag2–Ag2	107.52(12)
N21–Ag2–N2	88.91(14)
N21–Ag2–N22	177.33(17)
Re1–Se3–Re3	62.755(12)
Re3–Se1–Ag1	137.596(19)
C101–N11–Ag1	126.8(3)
C105–N11–Ag1	117.0(3)
C120–N12–Ag1	126.6(3)
C121–N12–Ag1	116.2(3)
C2–N2–Ag2	108.1(4)
C1–N1–Ag1	133.6(4)
C221–N22–Ag2	121.4(3)
C220–N22–Ag2	122.4(3)
C201–N21–Ag2	123.6(3)
C205–N21–Ag2	120.3(3)
N11–Ag1–Se1	91.43(11)
N11–Ag1–N12	172.51(15)
N11–Ag1–N1	93.55(14)
N12–Ag1–Se1	83.39(10)
N12–Ag1–N1	92.19(14)
N1–Ag1–Se1	172.23(10)

Table S8. Selected bond distances (Å) and angles (°) for **6**

Ag1–Ag1	3.3360(14)
Ag1–N1	2.622(8)

Ag1–N11	2.164(7)
Ag1–N12	2.165(7)
Ag2–N2	2.552(8)
Ag2–N21	2.193(7)
Ag2–S3	3.061(2)
Ag2–N22	2.183(7)
N1–Ag1–Ag1	131.66(17)
N11–Ag1–Ag1	74.1(2)
N11–Ag1–N1	90.6(3)
N11–Ag1–N12	177.0(3)
N12–Ag1–Ag1	108.3(2)
N12–Ag1–N1	89.0(3)
N21–Ag2–N2	93.1(3)
N22–Ag2–N2	92.5(3)
N22–Ag2–N21	174.3(3)
C1–N1–Ag1	105.7(6)
C101–N11–Ag1	121.8(6)
C105–N11–Ag1	121.4(6)
C120–N12–Ag1	120.3(6)
C121–N12–Ag1	123.4(6)
C2–N2–Ag2	131.7(7)
C201–N21–Ag2	117.1(6)
